# USMLE Step 2 Clinical Knowledge performance by disability, race, and ethnicity in U.S. medical students: A multi-site study

**DOI:** 10.1371/journal.pone.0349774

**Published:** 2026-05-21

**Authors:** Mytien Nguyen, Michael Kim, Karina Pereira-Lima, Tiffany Hodgens, Jordan Juliao, Elizabeth Holman, J. T. Sangsland, Dowin Boatright, Lisa M. Meeks

**Affiliations:** 1 Department of Immunobiology, Yale School of Medicine, New Haven, Connecticut, United States of America; 2 Department of Internal Medicine, University of Minnesota Medical School, Minneapolis, Minnesota, United States of America; 3 Department of Anesthesiology, University of Michigan Medical School, Ann Arbor, Michigan, United States of America; 4 University of California, Davis School of Medicine, Sacramento, California, United States of America; 5 Department of Medical Education, University of Illinois College of Medicine, Chicago, Illinois, United States of America; 6 University of Michigan Medical School, Ann Arbor, Michigan, United States of America; 7 Department of Emergency Medicine, New York University Grossman School of Medicine, New York, New York, United States of America; University of Science and Technology of Fujairah, YEMEN

## Abstract

**Importance:**

With the transition of USMLE Step 1 to pass/fail, USMLE Step 2 Clinical Knowledge (CK) has become a high-stakes component of residency selection. This shift raises concerns that increased reliance on Step 2 CK may exacerbate existing inequities, particularly for medical students with disabilities and those with intersecting marginalized identities.

**Objective:**

To examine differences in USMLE Step 2 CK performance by disability status, race, ethnicity, and their intersections among U.S. medical students.

**Methods:**

We conducted a cross-sectional analysis of the Pathways II dataset, comprising deidentified student-level data from nine U.S. MD-granting medical schools. Graduated students from the 2020–2023 cohorts were included. Medical students with disabilities were defined as those formally registered with institutional disability resource offices and were matched 1:2 with nondisabled peers by institution, gender, graduation cohort, and MCAT score. Race and ethnicity were categorized as White, Asian, underrepresented in medicine (URiM, including American Indian/Alaska Native, Black/African American, Hispanic/Latino, and Native Hawaiian/Pacific Islander), or Other. Disability categories included ADHD, learning, psychological, mobility/sensory, chronic health, or other. Multivariable linear regression models estimated adjusted Step 2 CK score differences by disability status, race, ethnicity, and their interactions, adjusting for MCAT score and clustering standard errors at the school level.

**Results:**

Among 1,350 students, 448 (33.2%) had a registered disability. The mean Step 2 CK score was 244.3 (SD, 15.1). Students with disabilities scored lower than nondisabled peers (mean 239.6 vs 246.7; adjusted β, −7.02; 95% CI, −8.76 to −5.28). Students with ADHD, learning, or psychological disabilities scored 9–11 points lower than nondisabled peers (P < .001). URiM students scored lower than White students overall (adjusted β, −8.50; 95% CI, −10.74 to −6.25). Intersectional analysis demonstrated that disparities were largest among URiM and Asian students with disabilities.

**Conclusions:**

Medical students with disabilities experience significant disparities in Step 2 CK performance, particularly those with cognitive-based disabilities and intersecting marginalized identities. As Step 2 CK plays a larger role in residency selection, addressing structural barriers in assessment and accommodation processes is critical to advancing equity.

## Introduction

The transition of the United States Medical Licensing Examination (USMLE) Step 1 to pass/fail has reshaped the residency selection process, with increased emphasis placed on USMLE Step 2 Clinical Knowledge (CK) as a high-stakes tool for screening residency applicants [1]. Prior research suggests that the USMLE Step examinations can foster “exam mania,” reflecting a widespread belief among students that performance on these exams is the primary determinant of their future careers [2]. This heightened perception of importance, coupled with the shift of evaluative weight to Step 2 CK, raises concerns within medical education about the potential amplification of existing inequities, particularly for students who encounter structural barriers within training and assessment systems.

Medical students with disabilities represent a growing proportion of U.S. medical trainees [3], and have been shown to experience disparities across multiple educational outcomes and opportunities, including standardized testing performance [4–6], academic progression [6–8], placement into honor societies [9], all with material consequences for residency match success [10].

Disparities in performance outcomes are frequently attributed to structural barriers related to the USMLE accommodation processes at the institutional, cultural and organizational levels [10–18]. At the institutional level, substantial variation in Step 1 accommodation request and approval rates across medical schools suggests the presence of school-specific barriers, with disability support and specialization that varies by institution [5,8,13–15]. These include limited institutional support [12–14] and insufficient disability resource professional expertise [5,13,15,16] leaving many students to navigate the USMLE accommodation process independently. Students also report inadequate documentation as a primary reason for not requesting accommodation [17] a challenge that often persists into high-stakes examinations [6,8,11,12,14]. Documentation gaps may further reflect limited access to the resources needed for updated diagnostic testing, as well as cultural, familial, or first-generation influences that discourage diagnosis and help-seeking in favor of resilience-based narratives [19–21]. Organizational barriers further complicate access, including prolonged decision timelines and differential approval rates by disability category [5,6,8,12]. Although recent work published by the NBME reports no barriers for medical students with diabetes [22], a comparatively straightforward and noncontroversial diagnosis, this pattern has not been observed for students with attention-deficit disorder, psychological disabilities, or those diagnosed after matriculation, who continue to encounter barriers to requesting accommodations on Step exams [5,6]. These barriers may be further compounded for students with intersecting marginalized identities, as minoritized Asian and underrepresented in medicine (URiM) medical students with disabilities report higher burnout [23] and discrimination [24], and are less likely to request accommodations on the Step 1 exam [5].

Despite the increasing importance of Step 2 CK in residency selection, data on score differences by disability status, race, ethnicity and their intersections remain limited. In this multi-institutional cohort study, we examined Step 2CK performance across disability, race, ethnicity, and intersecting identities.

## Methods

We conducted a cross-sectional analysis of the Pathways II dataset, which includes deidentified data from nine U.S. MD-granting medical schools collected between April 2023 and 2024, as previously described [6]. In brief, data were obtained from individual student records in collaboration with school administrators and disability resource professionals. Medical students with disabilities were defined as those formally registered with disability resource offices during enrollment, and all eligible students from four graduating cohorts (2020–2023) were included. To ensure confidentiality, institutions followed a strict protocol in which disability resource professionals entered de-identified data into a standardized spreadsheet, and de-identified datasets were securely transferred to the senior PI. Each medical student with a disability was matched with two nondisabled controls from the same institution to reduce confounding, based on gender, graduation cohort, and MCAT score (within ±6.5 points of the 2020–2021 national matriculating average). The ± 6.5-point MCAT caliper was selected to approximate one-half of one standard deviation of the national MCAT score distribution for the 2020–2021 matriculating cohorts, a threshold commonly used in caliper matching to balance precision of the match with the feasibility of identifying suitable controls across nine institutions and four graduation cohorts. Race/ethnicity was not included in matching as it was an independent variable of interest. In most cases (95%), matches were within the same cohort, with adjacent-year cohorts used when exact matches were unavailable. Race and ethnicity were categorized as White, Asian, URiM (American Indian/Alaska Native, Black/African American, Hispanic/Latino, and Native Hawaiian/Pacific Islander), or Other. Disability categories included ADHD, learning, psychological, mobility/sensory, chronic health, or other.

Descriptive statistics summarized mean Step 2CK scores by demographic characteristics. We employed two complementary analytic strategies to examine associations between disability status, race, ethnicity, and Step 2 CK scores, with all models adjusting for MCAT scores and using robust standard errors clustered at the medical school level. First, we fit a series of multivariable linear regression models that separately estimated adjusted Step 2 CK score differences by (a) disability status (disabled vs. nondisabled), (b) disability category (ADHD, learning, psychological, mobility/sensory, chronic health, other, vs. nondisabled), and (c) race and ethnicity (Asian, URiM, Other vs. White). To directly examine intersectional disparities, we then constructed a joint race/ethnicity-disability status variable (e.g., White, Nondisabled) and entered it into a regression model as a single categorical predictor with “White, Nondisabled” as the reference group. This intersectional approach was selected because our primary objective was to estimate the adjusted Step 2 CK score for each subgroup defined by the intersection of race/ethnicity and disability status, rather than to decompose independent main effects.

To formally test whether the association between disability status and Step 2 CK performance varied significantly by racial/ethnic group, we fit a separate multivariable linear regression model that included multiplicative interaction terms between race/ethnicity and disability status, along with their constituent main effects. This model provided inferential support for patterns observed in the intersectional subgroup analysis.

Variance inflation factors (VIFs) were assessed for all predictors in both model specifications; all VIF values were below 5, indicating that multicollinearity did not meaningfully influence coefficient estimates or standard errors. A 2-sided P < .05 was deemed statistically significant. The University of Michigan Institutional Review Board deemed this study exempt. Consent was waived for this study as data were deidentified.

## Results

A total of 1,350 medical students from nine U.S. MD-granting medical schools met inclusion criteria. Of these, 448 (33.2%) were formally registered with institutional disability resource offices and classified as medical students with disabilities, while 902 (66.8%) were matched nondisabled controls. Regarding race and ethnicity, 679 (50.3%) identified as White, 248 (18.4%) as Asian, 244 (18.1%) as URiM, and 179 (13.3%) as Other (Table 1). Among students with disabilities, the most prevalent disability categories were psychological (n = 145; 32.4%), ADHD (n = 89; 19.9%), chronic health (n = 84; 18.8%), learning (n = 49; 10.9%), other (n = 45; 10.0%), and mobility/sensory (n = 36; 8.0%).

**Table 1:** Mean and standard deviation of Step 2CK scores by demographics.

	N	Step2CK score Mean (SD)	
**Total**	**1,350**	**244.33 (15.10)**	
**Disability**			**<0.001**
No	902	246.70 (14.25)	
Yes	448	239.55 (15.65)	
**Disability category**			**<0.001**
Nondisabled	902	246.70 (14.25)	
ADHD	89	235.79 (15.88)	
Learning	49	235.63 (17.22)	
Psychological	145	236.85 (15.26)	
Mobility/Sensory	36	247.00 (11.15)	
Chronic Health	84	245.24 (13.26)	
Other	45	243.38 (16.60)	
**Race and ethnicity**			**<0.001**
White	679	246.20 (14.60)	
Asian	248	245.96 (15.03)	
URiM	244	237.78 (15.27)	
Other	179	243.88 (14.43)	
**Race, ethnicity, and disability intersection**			**<0.001**
White, Nondisabled	437	247.99 (13.73)	
Asian, Nondisabled	184	248.91 (14.14)	
URiM, Nondisabled	169	240.87 (14.62)	
Other, Nondisabled	112	246.85 (13.69)	
White, MSWD	242	242.98 (15.57)	
Asian, MSWD	64	237.47 (14.39)	
URiM, MSWD	75	230.83 (14.47)	
Other, MSWD	67	238.93 (14.38)	

*MSWD=medical student with disability.

### By overall disability and category

The overall mean Step 2CK score was 244.3 (SD: 15.1), with medical students with disabilities scoring 7-points lower than nondisabled students (239.6 vs 246.7; adjusted β: −7.02; 95%CI: −8.76 to −5.28). Scores varied by disability category (p < 0.001): students with ADHD, learning, or psychological disabilities scored 9- to 11-points lower than nondisabled peers (Table 1). Among cognitive-based disability categories, students with ADHD scored 11.3 points lower than nondisabled peers (adjusted β: −11.32; 95% CI: −14.60 to −8.03), students with learning disabilities scored 10.8 points lower (adjusted β: −10.82; 95% CI: −15.35 to −6.30), and students with psychological disabilities scored 9.6 points lower (adjusted β: −9.63; 95% CI: −12.28 to −6.99). In contrast, students with mobility/sensory disabilities did not differ significantly from nondisabled peers (adjusted β: 1.03; 95% CI: −3.96 to 6.03), nor did students with chronic health conditions (adjusted β: −1.48; 95% CI: −4.95 to 1.98) or those classified as “other” disability (adjusted β: −2.80; 95% CI: −7.17 to 1.56).

### By Race/Ethnicity

Step 2CK scores also differed by race and ethnicity. URiM students scored 8-points lower than White students (adjusted β: −8.50; 95%CI: −10.74 to −6.25, p < .001). Asian students did not differ significantly from White students (adjusted β: −0.45; 95% CI: −2.73 to 1.81), nor did students in the Other racial/ethnic category.

### By Race/Ethnicity and Disability

To examine how disability and race/ethnicity jointly shaped Step 2 CK performance, we fit an intersectional model using a combined race/ethnicity–disability status variable with White, nondisabled students as the reference group (Table 2). Among nondisabled students, URiM students scored 6.8 points lower than White nondisabled peers (adjusted β: −6.84; 95% CI: −9.49 to −4.19; p < .001), while Asian nondisabled students (adjusted β: 0.88; 95% CI: −1.74 to 3.50) and Other nondisabled students (adjusted β: −1.33; 95% CI: −4.42 to 1.75) did not differ significantly from the reference group. Among students with disabilities, all racial/ethnic subgroups scored significantly lower than White, nondisabled peers. White students with disabilities scored 4.7 points lower (adjusted β: −4.74; 95% CI: −7.09 to −2.39), while Asian students with disabilities scored 10.1 points lower (adjusted β: −10.10; 95% CI: −14.00 to −6.21), and URiM students with disabilities experienced the largest adjusted gap at 17.4 points lower (adjusted β: −17.36; 95% CI: −20.98 to −13.74). Students in the Other racial/ethnic category with disabilities scored 8.7 points lower (adjusted β: −8.73; 95% CI: −12.54 to −4.92).

**Table 2:** Association between disability status, race, ethnicity, and their intersections with Step 2 CK score.

	N	Model 1 Β (95% CI)	Model 2 Β (95% CI)
**Disability**			
No	902	*Ref*	
Yes	448	−7.02 (−8.76 to −5.28)	
**Disability category**			
Nondisabled	902	*Ref*	
ADHD	89	−11.32 (−14.6 to −8.03)	
Learning	49	−10.82 (−15.35 to −6.3)	
Psychological	145	−9.63 (−12.28 to −6.99)	
Mobility/Sensory	36	1.03 (−3.96 to 6.03)	
Chronic Health	84	−1.48 (−4.95 to 1.98)	
Other	45	−2.80 (−7.17 to 1.56)	
**Race and ethnicity**			
White	679	*Ref*	
Asian	248	−0.45 (−2.73 to 1.81)	
URiM	244	−8.50 (−10.74 to −6.25)	
Other	179	−2.48 (−5.00 to 0.04)	
Race, ethnicity, and disability intersection*			
White, Nondisabled	437		*Ref*
Asian, Nondisabled	184		0.88 (−1.74 to 3.5)
URiM, Nondisabled	169		−6.84 (−9.49 to −4.19)
Other, Nondisabled	112		−1.33 (−4.42 to 1.75)
White, MSWD	242		−4.74 (−7.09 to −2.39)
Asian, MSWD	64		−10.10 (−14.00 to −6.21)
URiM, MSWD	75		−17.36 (−20.98 to −13.74)
Other, MSWD	67		−8.73 (−12.54 to −4.92)

*Significant interaction terms for Asian and URiM (Asian p = 0.012, URiM p = 0.015).

To formally test whether the association between disability status and Step 2 CK performance differed by racial/ethnic group, we fit a separate model including multiplicative interaction terms between race/ethnicity and disability status. Significant interaction effects were observed for Asian students (interaction p = 0.012) and URiM students (interaction p = 0.015, Table 2), indicating that the disability-associated score gap was significantly larger among these groups than among White students.

## Discussion

In this multi-institutional cohort study of graduates from nine medical schools, medical students with disabilities scored significantly lower on USMLE Step 2 CK than their nondisabled peers, even after adjustment for prior academic performance. Differences were most pronounced among medical students reporting attention deficit hyperactivity disorder, learning and psychological disabilities, and were further amplified at the intersection of disability and race or ethnicity. URiM students with disabilities experienced the largest adjusted score gaps, followed by Asian medical students with disabilities.

These patterns are consistent with prior studies demonstrating greater academic burden among medical students with cognitive-based disabilities, such as ADHD, psychological and learning disabilities [4–7], and those identifying as Asian and URiM [5,7]. The magnitude of the observed disparities warrants consideration in terms of their practical significance for residency selection. In this sample, the overall mean Step 2 CK score was 244.3 (SD: 15.1); thus, the adjusted score differences of −7.02 points for medical students with disabilities overall, −11.32 for medical students with ADHD, and −17.36 for URiM students with disabilities represent approximately 0.5, 0.7, and 1.1 standard deviations of the overall score distribution, respectively. These are not trivial differences. In the context of residency selection, where programs increasingly rely on Step 2 CK scores for applicant screening, score shifts of this magnitude can meaningfully alter a student’s position relative to program-specific screening thresholds and competitive benchmarks, reducing the likelihood of receiving interview invitations, particularly in specialties where numeric scores are heavily weighted in initial application review.

Rather than pointing to a single explanatory pathway, the observed differences likely reflect the cumulative influence of structural and contextual factors operating across training and assessment environments. For medical students with intersecting marginalized identities, these factors may include differential access to, or utilization of, accommodations [25]; variability in institutional support [13,17,18]; and negative perceptions of learning environments [26] shaped by ableism [27] fear of bias [17], discrimination [24] often leading to burnout [23]. Medical students with cognitive-based disabilities may face additional challenges, including barriers to accommodations for high-stakes examinations such as requirements for updated neuropsychological documentation—a financial and time-intensive process [28]. Data from the 2025 AAMC graduation questionnaire suggests that 7.1% of medical students with disabilities do not request accommodations at their institution, because they “do not have documentation to support [their] request” [17]. In the context of Step examinations, lack of documentation and resources to obtain documentation may result in some students testing without accommodations or with accommodations that do not fully address functional limitations. Accordingly, observed score differences may reflect variation in access to the exam rather than differences in clinical knowledge or preparedness.

Finally, the amplified disparities observed among URiM and Asian medical students with disabilities in this study underscore the importance of adopting an intersectional lens when examining assessment outcomes. Prior research suggests that medical students with multiple marginalized identities report lower perceptions of inclusion, trust, and fairness within learning environments, as well as higher exposure to mistreatment and discrimination [23,24]. These contextual factors are known to influence academic performance and well-being and may interact with assessment structures in ways that compound disadvantage. Our findings suggest that disability-related disparities cannot be fully understood or addressed without attention to how race, ethnicity, and disability jointly shape students’ experiences within assessment and selection systems. Collectively, these findings suggest that observed disparities extend beyond individual preparation and may reflect modifiable structural conditions that cannot be fully understood or addressed without attention to how race, ethnicity, and disability jointly shape students’ experiences within assessment and selection systems.

Taken together, these results highlight the need to critically examine the role of Step 2 CK within residency selection, particularly as reliance on numeric score thresholds increase. While standardized examinations are often positioned as objective tools, persistent group-level differences raise concerns about construct-irrelevant variance introduced by structural barriers to access. Without parallel efforts to ensure equitable accommodation processes and supportive institutional practices, heightened emphasis on Step 2 CK may inadvertently perpetuate inequities that medical education has sought to mitigate.

This study has several limitations. First, Step 2 CK–specific accommodation data were not available, precluding direct examination of whether observed score differences reflect differential access to or use of testing accommodations. Second, although MCAT score was included as both a matching variable and a covariate in regression models, other academic performance indicators such as clerkship grades were not available in the Pathways II dataset and were therefore not included in matching or statistical adjustment. Residual confounding from these unmeasured variables cannot be excluded, and MCAT scores alone may not fully capture baseline academic ability. Notably, while Step 1 performance was available, it was intentionally excluded as prior work has demonstrated that Step 1 performance reflect disability-related disparities [3,5,6,8]; conditioning on a variable that may lie on the causal pathway between disability and Step 2 CK performance could introduce collider bias, distorting rather than clarifying the estimated associations. Third, participating medical schools self-selected into the Pathways II study, which may limit generalizability if these institutions are more engaged in disability inclusion efforts than non-participating schools. Finally, this analysis only collected data on graduated students, potentially failing to capture medical students with disabilities who were lost to attrition, creating results that reflect the best-case scenario. These limitations should be considered in the context of the study’s strengths, including the use of verified disability status, standardized score data, and a multi-institutional sample that allowed for intersectional analyses across disability, race, and ethnicity.

## Conclusion

As USMLE Step 2 CK assumes an increasingly important role in residency screening, score differentials of the magnitude observed in this study ranging from approximately one-half to over one standard deviation of the national score distribution may carry material consequences for medical students with disabilities, including reduced interview offers and downstream effects on match outcomes, across a range of specialties where Step 2 CK scores inform screening decisions. Because score thresholds used by residency programs vary widely by specialty and institution, the observed disparities have the potential to affect medical students differentially depending on their specialty of interest, compounding existing barriers to equitable workforce diversification.

The persistence of larger score gaps among minoritized medical students with disabilities suggests that structural disparities within assessment and selection systems remain inadequately addressed. Collectively, these findings argue for a re-examination of Step 2 CK’s role in residency evaluation to ensure that high-stakes testing reflects clinical readiness rather than unequal access to supports.

Future research should examine how access to Step 2 accommodations, institutional processes for supporting accommodations applications, and school-level supports for medical students with disabilities shape access and performance on high-stakes exams, especially for medical students with intersecting marginalized identities. Critically, future studies should investigate how the score differentials identified in this study translate into downstream consequences across the residency selection pathway. Qualitative and mixed-methods research should also be engaged as a means of exploring how residency program directors interpret and use Step 2 CK scores in the context of disability disclosure and holistic selection processes. These qualitative approaches would provide important context for understanding whether current selection practices inadvertently disadvantage applicants with disabilities. Finally, longitudinal studies examining whether Step 2 CK score differentials predict meaningful differences in residency performance, clinical competency, or patient outcomes are needed to assess whether the score gaps observed in this study reflect true differences in clinical readiness or, alternatively, represent construct-irrelevant variance attributable to structural barriers in assessment access.
